# Health system development and utilisation in Kuwait, 2011–2022: insights from national healthcare data

**DOI:** 10.3389/frhs.2026.1850596

**Published:** 2026-07-17

**Authors:** Sultan E. Alsalahi, Mohammad Almari, Lujain AlAbdulJaleel, Naser A. Albazzaz, Yasmin Al Sarraj, Hebatallah Subhi Akkad, Shahid Aziz, Abdulaziz Alhenaidi

**Affiliations:** 1 Ministry of Health, Kuwait City, Kuwait; 2 Department of Health Policy & Management, College of Public Health, Kuwait University, Kuwait City, Kuwait; 3 Department of Health Services Research & Policy, London School of Hygiene & Tropical Medicine, University of London, London, United Kingdom; 4 Dasman Diabetes Institute, Kuwait City, Kuwait; 5 Quality and Medical Cooperation Department, Medical Services Authority, Ministry of Defence, Kuwait City, Kuwait

**Keywords:** COVID-19 pandemic, health expenditure, healthcare utilisation, Kuwait, segmented regression, vaccination uptake, workforce

## Abstract

**Background:**

Despite substantial public investment, no study has examined Kuwait's health-system inputs and service utilisation or quantified COVID-19's impact across nationality strata.

**Methods:**

A retrospective interrupted time-series analysis examined 39 indicators from Kuwait Ministry of Health reports (2011–2022), stratified by nationality, covering infrastructure, expenditure, workforce, utilisation, and vaccination. Segmented regression with a 2019.5 breakpoint modelled the pre-pandemic trend (*β*_1_), pandemic-onset level shift (*β*_2_), and post-pandemic slope change (*β*_3_), fitted by ordinary least squares (OLS) and AR(1) generalised least squares (GLS) for all indicators, with the AR(1) estimates used where autocorrelation was detected.

**Results:**

Hospital beds rose pre-pandemic (*β*_1_ =  + 88.9/year, *p* < .001) and increased at onset (*β*_2_ =  + 842, +11.5%, *p* = .002), with no further change (*β*_3_, *p* = .28). Government salaries grew pre-pandemic (*β*_1_ = +KWD 61.2 million/year, *p* < .001), rose at onset (*β*_2_ = +KWD 95.8 million, +9.5%, *p* = .025), then decelerated (*β*_3_ = −KWD 71.4 million/year, *p* = .002). Kuwaiti nurses fell pre-pandemic (*β*_1_ = −20.6/year, *p* < .001) but recovered afterwards (*β*_3_ =  + 42.6/year, *p* = .013). Utilisation showed the sharpest disruption: Kuwaiti general-practitioner (GP) visits dropped at onset (*β*_2_ = −7.07 million, −68.3%, *p* < .001) before recovering (*β*_3_ =  + 1.40 million/year, *p* < .001), while non-Kuwaiti GP visits fell by 2.32 million (−48.6%, *p* = .012) without recovery. Kuwaiti diabetes-care visits declined at onset (−68,601, −17.0%, *p* = .006) then recovered (*β*_3_ =  + 25,919/year, *p* = .012). Non-Kuwaiti influenza vaccinations fell at onset (*β*_2_ = −106,731, *p* = .027) before recovering (*β*_3_ =  + 41,184/year, *p* = .044); meningitis and most routine vaccinations showed no significant change (*p* > .05).

**Conclusion:**

Kuwait's health system expanded over 2011–2022. COVID-19 caused significant utilisation disruption with substantial recovery, reflecting resilience. Nursing remains expatriate-dependent; sustained investment in primary and preventive care is critical.

## Introduction

Kuwait, a high-income country in the Gulf Cooperation Council (GCC) (1), has achieved notable milestones in developing its national healthcare system (2). The country provides publicly funded, free-at-the-point-of-use health services to citizens, while expatriates access healthcare through a parallel system in government health facilities, regulated by mandatory insurance schemes (2, 3). Kuwait has a modern healthcare system with relatively strong health indicators, including a life expectancy of 80 years in 2022 (1), a maternal mortality ratio of 8 per 100,000 live births, 100% coverage of skilled birth attendance, and an under-five mortality rate of 8.1 deaths per 1,000 live births in 2023 (4). According to the Public Authority for Civil Information (PACI), Kuwait's population reached about 5.2 million in December 2025, with expatriates accounting for 3.6 million, of whom about 3.1 million are male (5). The Ministry of Health (MOH) is the main provider of health services, structured into three tiers: primary, secondary, and tertiary care. These services are available to all residents across Kuwait's six regions: Al-Assimah (Capital), Hawalli, Al-Ahmadi, Al-Jahra, Al-Farwaniya, and Mubarak Al Kabeer. Each region has several clinics and at least one general hospital offering preventive, curative, and rehabilitative services.

As Kuwait grows economically (6), and as in other GCC countries (7), the burden of non-communicable diseases (NCDs) has increased significantly and has become the leading cause of mortality (8). The World Health Organisation (WHO) estimates that about one in five adults in Kuwait dies from NCDs before age 70 (9). Given the country's disease patterns and high prevalence of NCDs, transitioning from curative to preventative medicine is essential. Additionally, population growth and ageing significantly increase demand for health services and pose challenges for healthcare systems. Coupled with rising life expectancy, these trends highlight the need for a resilient, efficient, and sustainable healthcare system. Additionally, similar to other GCC countries, Kuwait is facing challenges related to rising healthcare costs (10, 11) and a reliance on non-national healthcare workers (12, 13).

Kuwait's healthcare facilities heavily depend on an expatriate workforce to meet the demand for skilled professionals (13). Although the government has implemented the “Kuwaitisation” policy to reduce reliance on foreign workers, the continued employment of non-Kuwaiti staff remains essential. This situation highlights the ongoing challenge of maintaining a high standard of healthcare services in the country.

Global healthcare infrastructure has progressed significantly. According to the WHO, most countries have expanded their healthcare coverage and reduced the financial hardship associated with healthcare costs (14). Countries have developed their infrastructure by improving buildings (15, 16), equipment (17), access to services (18), and digital infrastructure (19, 20). Likewise, GCC countries have expanded their healthcare infrastructure (10, 21, 22). As a result of such investment and other factors, such as ageing and NCDs (23, 24), healthcare spending has risen globally (25–27) and in the GCC countries (22, 28, 29). Regarding the health workforce, there has been an increase in numbers (30); however, there remains an unequal distribution between high- and low-income countries (30, 31), workforce shortages (32), and migration (33, 34). Despite the expansion of educational (13) and nationalisation programmes (35, 36), GCC countries still depend on expatriate health workers (13, 21), especially nurses (37).

In terms of healthcare utilisation, global healthcare utilisation rates have increased over the last two decades, mainly due to demographic changes and improved access to services (38–41). However, utilisation rates were impacted worldwide as a consequence of the COVID-19 pandemic. Several studies in the literature have reported a reduction in healthcare utilisation during the pandemic, particularly for less severe or elective conditions (42, 43). The migrant or non-national population was more affected by the pandemic's impact on healthcare utilisation, as studies showed they experienced greater reductions in utilisation (44, 45). While recovery for the utilisation was observed in some services, others did not recover, especially among the disadvantaged population (46). As observed globally, the utilisation of healthcare services in the GCC countries decreased during the pandemic, as shown in studies from the United Arab Emirates (47), Qatar (48), and Saudi Arabia (49). However, there is limited data on differences in healthcare utilisation between nationals and non-nationals in the GCC, including Kuwait.

While few studies have examined health expenditure in Kuwait (50), to our knowledge, none have examined it in detail, nor have they examined infrastructure development in the Kuwaiti health system. Regarding the Kuwaiti health workforce, previous studies were conducted more than 15 years ago (51–53). In terms of service utilisation, a study was found that examined healthcare utilisation with a focus on NCDs (8); however, to our knowledge, none has examined healthcare utilisation in detail or from the lens of national vs. non-national populations. This research provides a comprehensive overview of Kuwait's healthcare system and its evolution from 2011 to 2022 and evaluates the impact of the COVID-19 pandemic on that trajectory.

## Methods

### Study design and objectives

This is a retrospective, longitudinal study using an interrupted time-series design that employed and extracted publicly available aggregated data to examine trends in workforce, service utilisation, infrastructure, financial indicators, and vaccinations. Specifically, the study has three objectives:

(i) To describe absolute and relative changes across the full 2011–2022 period using percentage-change and time-trend analysis.
(ii) To estimate the effect of the COVID-19 pandemic on each of these domains using segmented linear regression with a 2019.5 breakpoint.
(iii) To compare these trends between Kuwaiti and non-Kuwaiti populations across all five domains.

### Data sources and pre-processing

Reports were available in soft versions, and data were extracted manually into Microsoft Excel for aggregation. Each indicator was extracted once per year directly from the corresponding MOH Annual Health Report; therefore, no cross-source overlap or de-duplication was required. The dataset was complete, with a reported value available for every indicator in all study years (2011–2022); no data were missing, and no imputation was therefore performed. The full list of study variables, grouped by domain, with variable type, unit, data source, and nationality stratification, is presented in Table 1.

- Workforce: Information was extracted from the workforce sections of annual health reports. The PACI (5) website was used to obtain information on Kuwait's population. Workforce density per 1,000 was calculated using the following formula: [(total number of professionals/total population) × 1,000].
- Service Utilisation: Services were obtained from the services section of the annual health report, which mentioned the number of services provided by general and tertiary hospitals and primary healthcare centres.
- Infrastructure and Financial Indicators: Information was obtained by extracting data on the number of facilities constructed and financial indicators between 2011 and 2022.

**Table 1:** Study variables and classification.

Domain	Variable	Type	Unit	Source	By nat.
Infrastructure	General & specialty hospitals; private hospitals; hospital beds; primary healthcare (PHC) centres; child-care, dental, diabetes clinics	Count	*n* facilities; *n* beds	MOH Annual Health Report 2011–2022	No
Expenditure	Approved budget; executed budget; salaries & benefits; drugs & equipment; per-capita spending; MOH share of national budget	Monetary; ratio	KWD; %	MOH Annual Health Report 2011–2022	No
Workforce	Physicians; nurses; pharmacists; dentists; medical technicians; administrators; service personnel; vocational assistants	Count; density	*n*; per 1,000 pop.	MOH Annual Health Report 2011–2022;	Yes
Service utilisation	General-hospital discharges; tertiary-care discharges; GP visits; child-care visits; dental-care visits; diabetes-care visits	Count	*n* visits/*n* discharges	MOH Annual Health Report 2011–2022	Yes
Vaccination	Hepatitis B; meningitis; influenza; MMR (dose 1, dose 2)	Count	*n* doses administered	MOH Annual Health Report 2011–2022	Yes

MOH, Ministry of Health, Kuwait. “By nat.” indicates whether the indicator is stratified by Kuwaiti vs. non-Kuwaiti population. Pre-pandemic period covers 2011–2019 (9 annual observations); post-pandemic period covers 2020–2022 (3 annual observations); segmented-regression breakpoint placed at 2019.5.

For comparative purposes, percentage changes between baseline (2011) and endline (2022) were computed alongside regression-derived annual change estimates to capture both absolute and relative changes over time. The percentage change between two time points was computed as [(Yfinal − Ybaseline)/Ybaseline] × 100.

### Model specification and segmented regression analysis

Model specification followed *a priori* protocol rather than a data-driven search to avoid overfitting in short series. Interrupted time-series analysis with a single breakpoint is the recommended quasi-experimental design for evaluating system-level shocks when randomisation is not possible (54).

**Figure 1:**
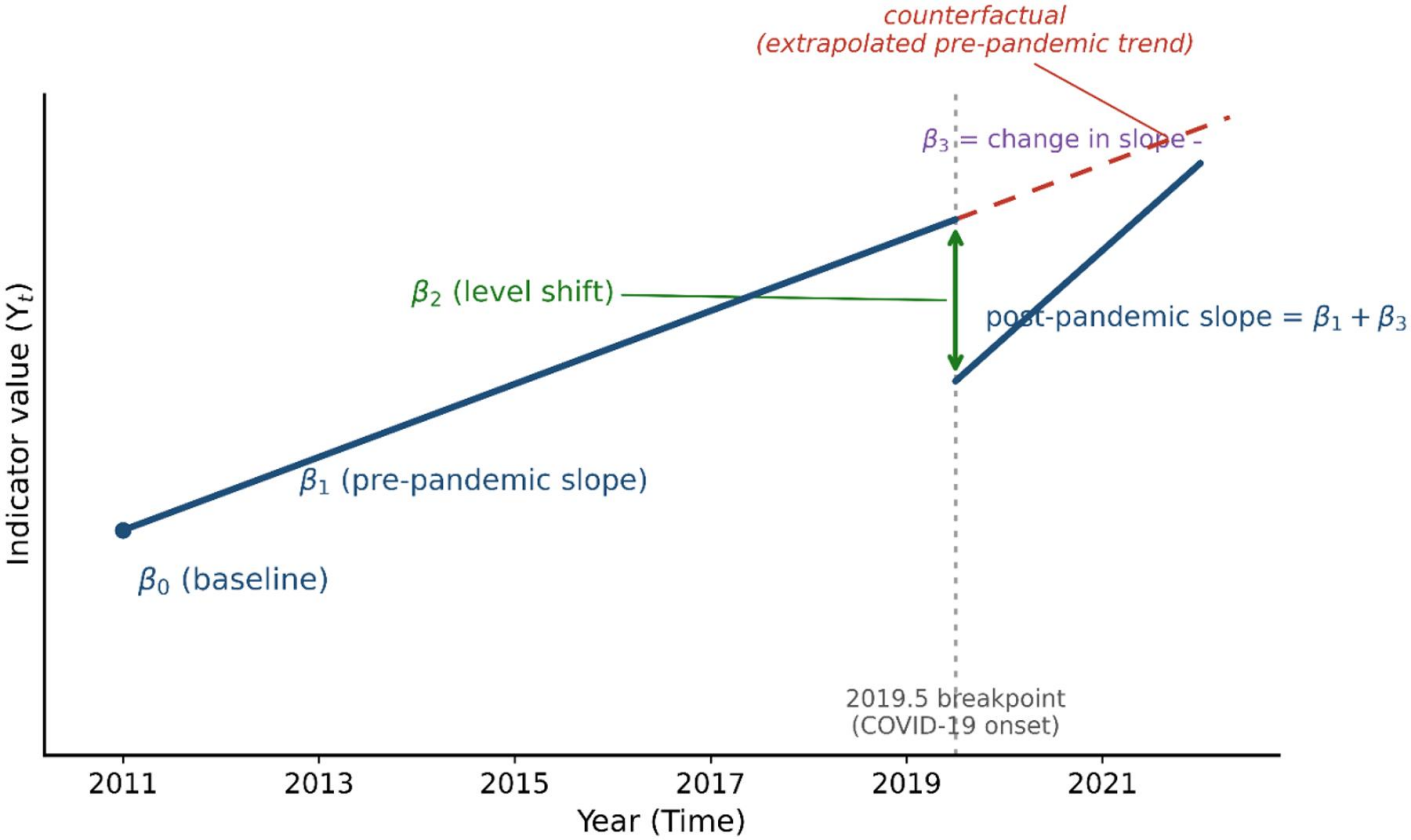
Conceptual representation of the four-parameter segmented interrupted-time-series regression model. *β*_0_ = baseline level at the start of the time series; *β*_1_ = pre-pandemic annual slope; *β*_2_ = immediate level shift at the 2019.5 breakpoint (the “shock” effect of pandemic onset); *β*_3_ = change in slope post-2020, so the post-pandemic annual slope equals (*β*_1_ + β_3_). The dashed line extrapolates the pre-pandemic counterfactual trend.

The general four-parameter segmented regression model, specified ex ante as: Y = *β*₀ + *β*₁(Time) + *β*₂(Pandemic) +  *β*₃(TimeAfterPandemic) + *ε*t. A conceptual representation is provided in Figure 1.

- Yt is the outcome variable at time t.
- Timet represents the continuous time variable indicating the number of years since baseline (2011 = 0).
- Pandemict is a binary indicator (0 before 2020, 1 from 2020 onwards).
- TimeAfterPandemict represents the number of years after the breakpoint.
- *β*_0_ identifies the baseline level.
- *β*_1_ estimates the pre-2020 trend.
- *β*_2_ estimates the immediate level shift at the 2019.5 breakpoint.
- *β*_3_ estimates the change in slope after the 2019.5 breakpoint.

### Time-series assumptions and model diagnostics

Outliers were screened using standardised residuals (|*Z*| > 3); the maximum studentised residual per indicator is reported in Supplementary Table S1. Residual normality was assessed by Q–Q plots and the Shapiro–Wilk test (Supplementary Table S1), stationarity by the Augmented Dickey–Fuller test in levels and first differences (Supplementary Table S2), and autocorrelation by the Durbin–Watson statistic (Supplementary Table S1).

All 39 indicators were re-estimated by GLS with AR(1). OLS and AR(1) estimates, standard errors, *p*-values, and 95% confidence intervals are reported side-by-side in Supplementary Table S3 [S3a, OLS; S3b, AR(1) GLS]; OLS-based AIC (Akaike information criterion) and BIC (Bayesian information criterion) are reported in Supplementary Table S1. Where the two diverged in magnitude or significance, the more conservative AR(1) estimate was preferred; for indicators with non-normal or borderline residuals (Shapiro–Wilk *p* < .10), inference was additionally supported by 1,000-replicate bootstrap BCa 95% confidence intervals (Supplementary Table S4).

### Data-point asymmetry

Reports were available only through 2022 (the 2023–2024 yearbooks are unreleased), giving an asymmetric series of 9 pre-pandemic (2011–2019) and 3 post-pandemic (2020–2022) annual points.

### Statistical analysis

All statistical analyses, including the segmented regression models, residual diagnostics, the AR(1) GLS re-estimation, and the bootstrap confidence intervals, were performed in IBM SPSS Statistics version 30 (IBM Corp., Armonk, NY, USA). Augmented Dickey–Fuller stationarity tests were performed in Python version 3.13 using the statsmodels package (version 0.14). All charts and figures were produced in GraphPad Prism version 10.6.0 (GraphPad Software, San Diego, CA, USA). Statistical significance was assessed at *α* = .05, with exact *p*-values and 95% confidence intervals reported for all estimates.

## Results

### Infrastructure development and health expenditure

Between 2011 and 2022, the general and tertiary hospitals grew from 14 to 20 (+43%). Similarly, the number of hospital beds, both general and tertiary, has grown from 6,703 to 8,735 (+30%) (Table 2) (55, 56). The number of private hospitals increased from 8 to 13 (+63%) between 2011 and 2022. The number of PHC and childcare clinics each rose, with 19 clinics opening (+21%). Also, diabetes care clinics and dental care clinics grew from 64 to 80 to 102 (+59%) and 90 (+13%), respectively (Table 2).

**Table 2:** Infrastructure facilities count in 2011 and 2022.

Facility	2011	2022	Change %
General and specialised hospitals	14	20	43%
Hospital Beds	6,703	8,735	30%
Primary Health Care Centers	92	111	21%
Child Care Clinics	92	111	21%
Dental Care clinics	80	90	13%
Diabetes Care clinics	64	102	59%
Private Hospitals	8	13	63%

#### Segmented regression

Pre-pandemic (2011–2019), hospital beds grew by *β*_1_ = 88.9 beds/year (SE = 14.0; *p* < .001) and primary healthcare centres by *β*_1_ = 1.13/year (SE = 0.24; *p* = .001). At the 2020 breakpoint, hospital beds showed a significant positive level shift (*β*_2_ =  + 841.8 beds; SE = 178.4; *p* = .002) corresponding to the opening of new hospital capacity during the pandemic response, while PHC centres showed a small negative level shift (*β*_2_ = −9.6; *p* = .013). Post-pandemic (2020–2022), the PHC slope increased significantly (*β*_3_ =  + 5.37/year; *p* = .004), and bed growth continued, but the slope change was not statistically distinct from the pre-pandemic trend (*β*_3_ =  + 90.1/year; *p* = .28). General/specialty and private hospital counts showed no significant level shifts at 2020 (Supplementary Table S3).

All financial indicators increased from 2011 to 2022 (Table 3). The Kuwait government's approved budget for the MOH was raised by 175%, from 1.2 KWD billion to 3.3 KWD billion, and the executed budget by 209%, from 1.0 KWD billion to 3.2 KWD billion. The MOH's share of the country's total budget increased by 112% during the same period, reaching 14%. Per capita spending and workforce salaries have also risen significantly (by 90% and 95%, respectively). Spending on drugs and equipment has surged by 152%, from 207 million KWD to 521 million KWD (Table 3).

**Table 3:** Financial aspect between 2011 and 2022.

Financial aspect	2011	2022	Change %
Approved budgets (millions KWD)	1,197	3,293	175%
Executed budgets (millions KWD)	1,040	3,214	209%
Per capita spending (KWD)	263	499	90%
Salaries & benefits (millions KWD)	556	1,086	95%
Drugs and equipment (millions KWD)	207	521	152%
% of M.O.H. budget from country budget	6.6%	14%	112%

#### Segmented regression

Pre-pandemic (2011–2019), government health-sector salaries grew by *β*_1_ = KWD 61.2 million/year (SE = 2.73; *p* < .001) and drugs & equipment spending by *β*_1_ = KWD 40.5 million/year (SE = 4.53; *p* < .001). At the 2020 breakpoint, salaries showed a positive level shift (*β*_2_ = +KWD 95.8 million; SE = 34.9; *p* = .025), reflecting the COVID-19 emergency hiring and risk-allowance payments; drugs & equipment and the executed budget showed no significant level shift. Post-pandemic (2020–2022), salary growth decelerated significantly (*β*_3_ = −KWD 71.4 million/year; SE = 15.2; *p* = .002), while drugs & equipment continued without a significant slope change (Supplementary Table S3).

### Workforce demographics

**Figure 2:**
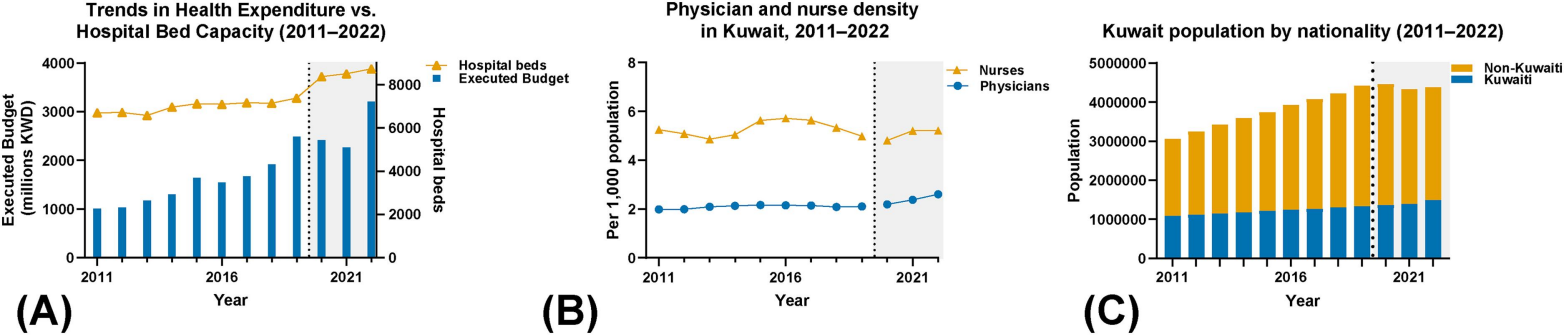
Shows trends in health expenditure vs. hospital bed capacity in Kuwait from 2011 to 2022 (panel **A**), physician and nurse density over the same period (panel **B**), and Kuwait's population by nationality (panel **C**).

Between 2011 and 2022, the number of Kuwaiti and non-Kuwaiti healthcare professionals increased noticeably (Table 4). K healthcare professional recruitment showed increases in workforce density among dentists (+80%), physicians (+33%), medical technicians (+3%), and pharmacists (+66%). However, nurses (−37%) and all other non-medical professionals, such as service personnel (−35%), administrators (−20%), and vocational assistants (−57%), have all experienced workforce reductions (Table 4). Furthermore, non-Kuwaiti workforce density has also shown similar trends with physicians (Figure 2), dentists, pharmacists and medical technicians showing increases in workforce density (+30%, +10%, +59%, +30%, respectively) in addition to a modest increase in density of nurses over the 11-year period (2%) (Figure 2). Similarly, non-Kuwaiti non-medical technicians (−38%), vocational assistants (−27%), service personnel (−34%) and administrators (−2%) have all shown reductions in workforce density (Table 4). By 2022, Kuwaitis constituted approximately 75% of the dental workforce and the majority of administrators, but remained a minority among physicians (41%), pharmacists, and medical technicians, and only approximately 5% of nurses. Segmented regression showed that both Kuwaiti and non-Kuwaiti physicians, pharmacists, dentists, and medical technicians experienced increases pre-2020 and accelerated increases post-2020. However, Kuwaiti nurses showed a significant pre-2020 decline, which turned into a positive trend post-2020, while non-Kuwaiti nurses showed pre-2020 increases but a non-significant decline post-2020 (Supplementary Table S3).

**Table 4:** Healthcare workforce numbers in 2011 and 2022.

Density (per 1,000)	Kuwaitis			Non-Kuwaitis			Total (2022)
	2011	2022	Change %	2011	2022	Change %	
Physicians	0.80	1.06	33%	1.19	1.54	30%	2.60
Dentists	0.27	0.48	80%	0.15	0.16	10%	0.63
Nurses	0.40	0.26	−37%	4.85	4.96	2%	5.22
Pharmacists	0.15	0.25	66%	0.18	0.29	59%	0.54
Medical tech	1.51	1.55	3%	1.11	1.45	30%	2.99
Non-medical tech	0.71	0.58	−18%	0.11	0.05	−57%	0.63
Vocational assistants	0.01	0.00	−57%	0.20	0.14	−27%	0.15
Service personnel	0.08	0.05	−35%	0.05	0.03	−34%	0.08
Administrators	2.92	2.32	−20%	0.23	0.23	−2%	2.55

#### Segmented regression

Pre-pandemic (2011–2019), Kuwaiti physicians grew by *β*_1_ =  + 168.5/year (*p* < .001), Kuwaiti dentists by *β*_1_ =  + 90.2/year (*p* < .001), Kuwaiti pharmacists by *β*_1_ =  + 44.9/year (*p* < .001), and Kuwaiti medical technicians by *β*_1_ =  + 180.7/year (*p* < .001); non-Kuwaiti physicians, pharmacists, dentists and medical technicians showed similar positive pre-pandemic trends. Kuwaiti nurses declined steadily pre-pandemic (*β*_1_ = −20.6/year; *p* < .001), while non-Kuwaiti nurses grew (*β*_1_ =  + 1,004.3/year; *p* < .001). At the 2020 breakpoint, no workforce cadre showed a statistically significant level shift in the Kuwaiti population, but non-Kuwaiti pharmacists (*β*_2_ = −306.0; *p* = .018) and non-Kuwaiti medical technicians (*β*_2_ = −1,322.8; *p* = .006) showed significant negative level shifts. Post-pandemic (2020–2022), the slope-change parameter *β*_3_ was significantly positive for Kuwaiti physicians (+132.0/year, *p* < .001), Kuwaiti pharmacists (+63.6/year, *p* = .003), Kuwaiti dentists (+109.3/year, *p* < .001), Kuwaiti nurses (+42.6/year, *p* = .013), non-Kuwaiti pharmacists (+208.6/year, *p* = .002) and non-Kuwaiti medical technicians (+1,028.9/year, *p* < .001) (Supplementary Table S3).

### Healthcare services utilisation

Between 2011 and 2022, the overall utilisation of healthcare services in MOH showed variable demand across service types, ranging from Kuwaitis to non-Kuwaitis. While the top 100 surgeries have increased by 12% among Kuwaitis, non-Kuwaitis have seen a slight 1% increase. Furthermore, the number of Kuwaitis discharged from general hospitals increased by 9%, but decreased from tertiary hospitals by 20%. On the contrary, the number of non-Kuwaiti discharges from general hospitals has declined by 8% and 36% in tertiary hospitals. Visits to general hospitals’ outpatient clinics by Kuwaitis and non-Kuwaitis have increased (+122%, +36% respectively), while visits to tertiary hospitals have decreased (−2%, −26% respectively). In the same period, Kuwaitis' visits to General Practitioners (GPs) increased by 16%, while non-Kuwaitis' visits decreased by 12%. Both Kuwaiti and non-Kuwaiti visits to child-care services (−28% and −30%) and dental services (−11% and −3%) decreased, while diabetes-care visits increased (+20% and +30%, respectively).

#### Segmented regression

**Figure 3:**
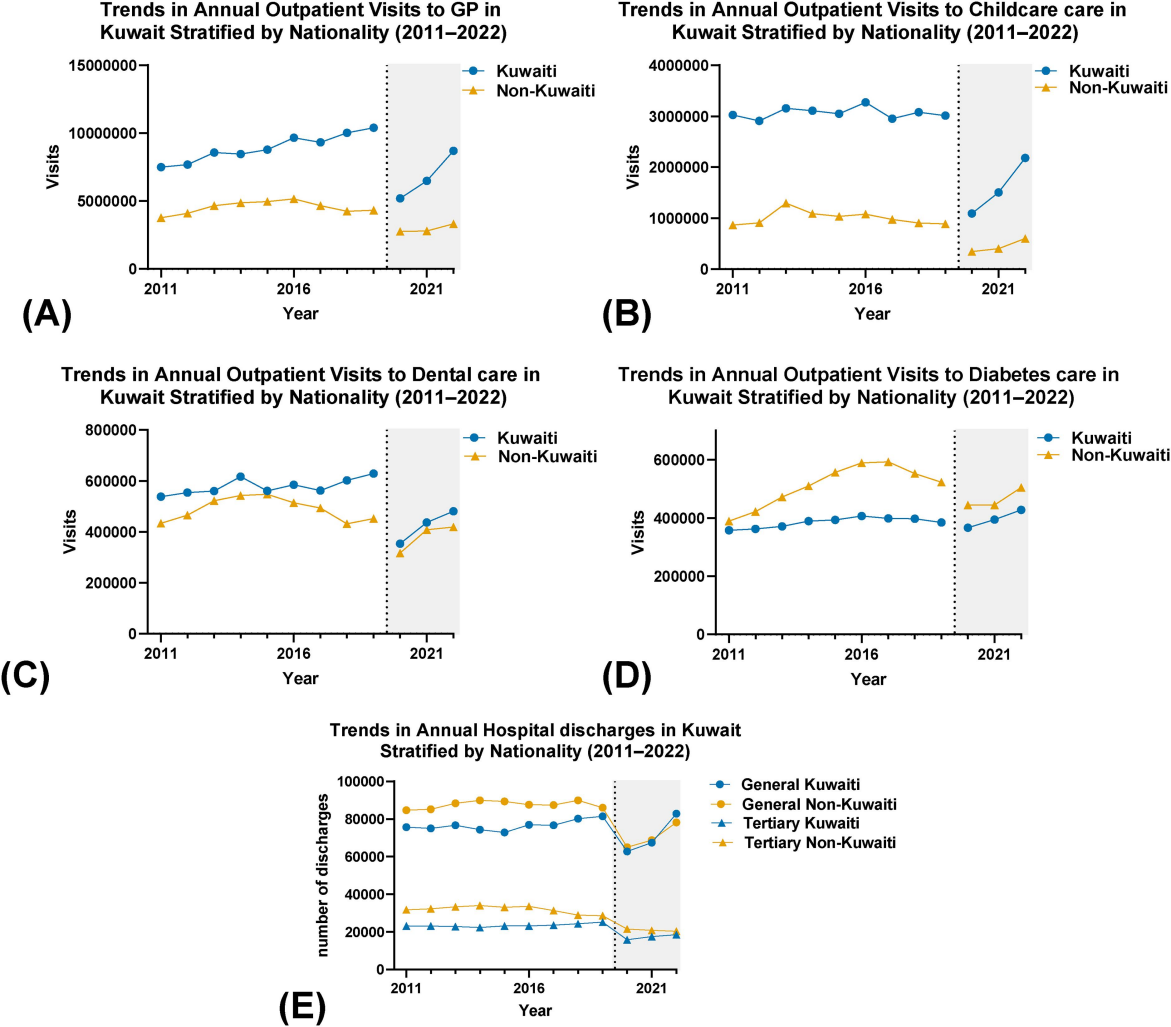
Shows annual outpatient visits to general practice (panel **A**), childcare services (panel **B**), dental care (panel **C**), diabetes care (panel **D**) and hospital discharges (panel **E**) in Kuwait stratified by nationality between 2011 and 2022.

Pre-pandemic (2011–2019), Kuwaiti tertiary-care discharges grew modestly (*β*_1_ =  + 230.9/year; SE = 71.0; *p* = .012), Kuwaiti dental visits grew by *β*_1_ =  + 7,956/year (*p* = .027), and diabetes-care visits grew in both populations (Kuwaiti *β*_1_ =  + 4,727/year, *p* = .011; non-Kuwaiti *β*_1_ =  + 20,773/year, *p* = .007). At the 2020 breakpoint, large negative level shifts were observed for Kuwaiti GP visits (*β*_2_ = −7,071,803; *p* < .001), Kuwaiti child-care visits (*β*_2_ = −2,574,075; *p* < .001), Kuwaiti dental-care visits (*β*_2_ = −314,062; *p* < .001), tertiary-care discharges in both populations (Kuwaiti *β*_2_ = −9,766, *p* < .001; non-Kuwaiti *β*_2_ = −8,050, *p* = .013), non-Kuwaiti dental-care visits (*β*_2_ = −202,750; *p* = .029), Kuwaiti diabetes-care visits (*β*_2_ = −68,601; *p* = .006), and non-Kuwaiti diabetes-care visits (*β*_2_ = −190,980; *p* = .031). Post-pandemic (2020–2022), recovery slopes were positive and significant for Kuwaiti GP visits (*β*_3_ =  + 1.40 million/year; *p* < .001), Kuwaiti child-care visits (*β*_3_ =  + 541,468/year; *p* < .001), Kuwaiti dental-care visits (*β*_3_ =  + 55,697/year; *p* = .009), Kuwaiti tertiary-care discharges (*β*_3_ =  + 1,123/year; *p* = .022), and Kuwaiti diabetes-care visits (*β*_3_ =  + 25,919/year; *p* = .012). Recovery in the non-Kuwaiti population was less consistent, with diabetes-care visits showing no significant rebound (*β*_3_ =  + 9,496; *p* = .77) (Supplementary Table S3) (Figure 3).

### Vaccinations

**Figure 4:**
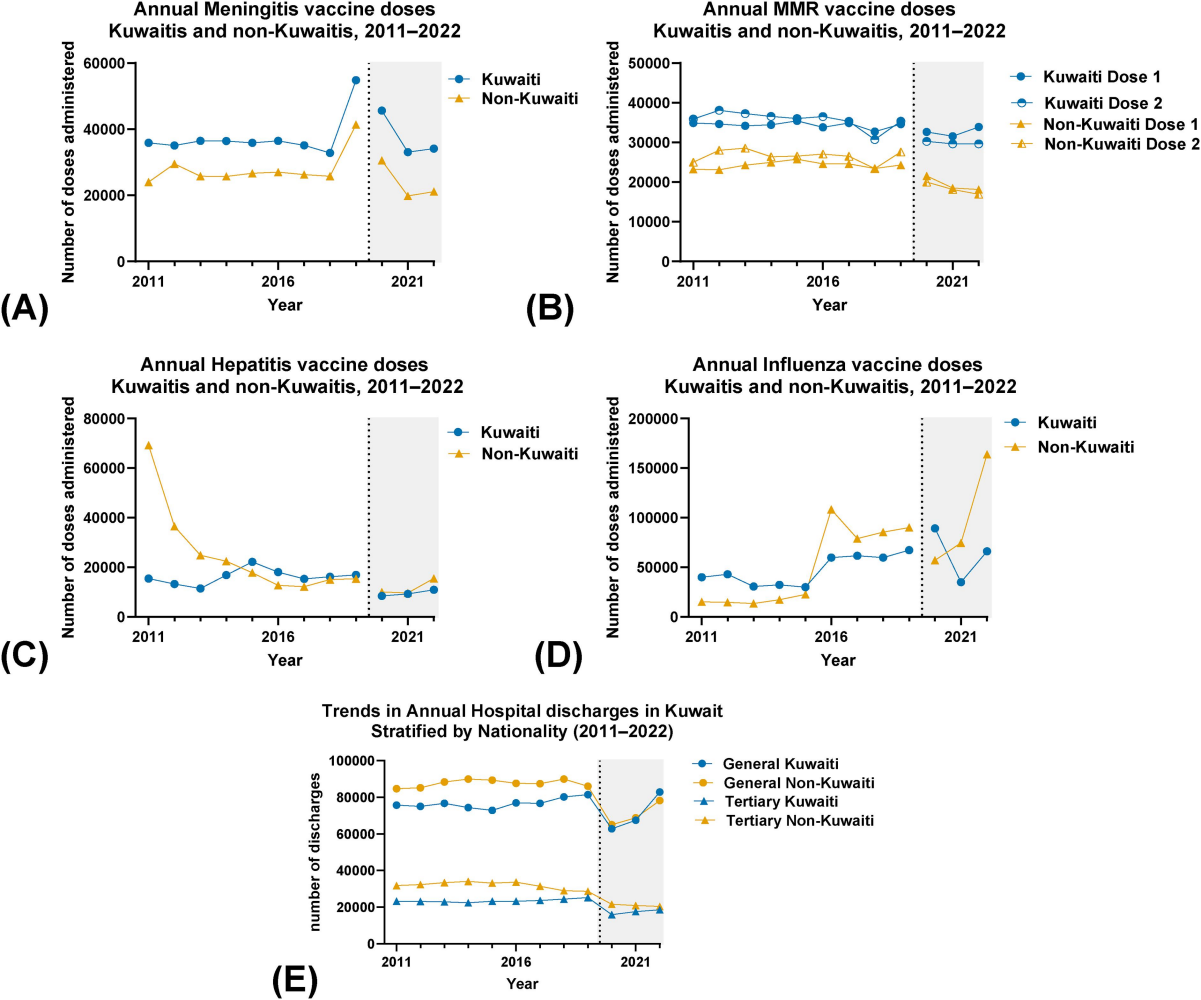
Shows annual doses of meningitis vaccine (panel **A**), measles–mumps–rubella vaccine dose 1 and dose 2 (panel **B**), hepatitis B vaccine (panel **C**), and influenza vaccine (panel **D**) administered to Kuwaitis and non-Kuwaitis between 2011 and 2022.

Disparate vaccination trends are observed according to vaccine type and nationality. Kuwaitis increased their uptake of the influenza vaccine by 66%; however, the uptake of the Measles, Mumps, and Rubella (MMR) vaccine (−10%) and Hepatitis B (−84%) has decreased. In the non-Kuwaiti population, the influenza vaccine and MMR vaccine uptake increased by 982% and 47%, respectively. Meanwhile, the uptake of non-Kuwaiti (−12%) and Kuwaiti (−5%) meningitis vaccination decreased while non-Kuwaiti hepatitis B uptake was essentially unchanged across the period (+0.6%) despite a significant transient drop at the 2020 pandemic onset. Figure 4 depicts the annual number of vaccine shots administered from the period of 2011 to 2022 (Supplementary Table S3).

#### Segmented regression

Pre-pandemic (2011–2019), influenza-vaccine uptake grew in both populations (Kuwaiti *β*_1_ =  + 4,162/year, *p* = .074; non-Kuwaiti *β*_1_ =  + 12,237/year, *p* = .004), while Hepatitis B uptake declined among Kuwaitis (*β*_1_ = −5,246/year; *p* = .007). At the 2020 breakpoint, statistically significant negative level shifts were observed for non-Kuwaiti influenza uptake (*β*_2_ = −106,731; *p* = .027) and non-Kuwaiti hepatitis B uptake (*β*_2_ = −11,465; *p* = .046); the remaining vaccinations showed no significant level shift. Post-pandemic (2020–2022), the only statistically significant slope changes were a recovery in non-Kuwaiti influenza uptake [*β*_3_ =  + 41,184/year; *p* = .044; under AR(1) GLS the estimate was comparable in magnitude but did not reach significance, *p* = .050] and a numerical decline in non-Kuwaiti MMR uptake (*β*_3_ = −3,226/year; *p* = .059). Meningitis-vaccine uptake showed no significant pre-pandemic trend, level shift at 2020, or post-pandemic slope change in either population (Supplementary Table S3).

## Discussion

The study assessed the Kuwaiti healthcare system retrospectively from 2011 to 2022. Findings suggest that the Kuwait MOH has shown robust progress in expanding health infrastructure, workforce, and healthcare services. However, expansions of healthcare services and health infrastructure have come at substantial government expenditure, accounting for a substantial share of the annual national budget.

### Infrastructure development and health expenditure

Kuwait's health infrastructure has expanded with the construction of mega hospitals and healthcare centres. Between 2011 and 2022, two general and four tertiary hospitals were constructed, reflecting the government's commitment to expanding healthcare capacity. The newly built hospitals have provided 2,000 beds, representing a 30% increase (from 6,703 to 8,735) during this study period. The segmented regression analysis indicates that this expansion was concentrated at the pandemic onset rather than representing a sustained acceleration in the underlying rate of growth, bed capacity showed a significant immediate level shift in 2020 (*β*_2_ =  + 842 beds; *p* = .002), while the change in the post-2020 annual rate of expansion was not statistically significant (*β*_3_ =  + 90 beds/year; *p* = .28). A comparable pattern was observed for the executed budget, which continued its strong pre-pandemic growth (*β*_1_ =  + 163 million KWD/year; *p* < .001) without a statistically significant change in level or slope at 2020. This reflects the government's commitment to strengthening the resilience of Kuwait's health system. Similarly, there were expansions in primary care services, with 19 PHCs added during this time. These expansions demonstrate national efforts to maximise access to healthcare and response to the rising burden of NCDs, in line with global trends in healthcare spending and capital expansion (56, 57).

Regarding financial aspects, one-half of the budget is allocated to salaries, drugs, and equipment, with over 1.6 billion KWD spent under the approved 2022 budget. These figures illustrate the government's intention to prioritise health as a national concern, particularly by increasing the health share of the national budget by more than two-fold (6.6% in 2011 vs. 14% in 2022), which is higher than neighbouring GCC countries of national budgets (Saudi Arabia 12.8%, Qatar 7.4%, Oman 8.3%, Bahrain 8.6%, United Arab Emirates 12.1%) (57). The increase in health financing may be attributed to multiple factors. Population growth (5) and the rise in NCDs in Kuwait over the past decade (8) have increased pressure on healthcare services, necessitating higher health expenditure. Another factor that could contribute to the increase in health expenditure is the cost of prescription drugs and medical equipment (58). Global market dynamics due to the existence of a monopoly, high costs of development of prescribed drugs, and the seriousness of the diseases all contribute to the high costs of prescription drugs (58).

### Workforce

The health workforce in Kuwait has shown increases in recruitment numbers from 2011 to 2022, while segmented regression analysis has revealed efforts of “Kuwaitisation” of the health workforce among several professional medical cadres. Indeed, the recruitment of both Kuwaiti and non-Kuwaiti physicians, dentists, medical technicians and pharmacists has shown a growth trajectory post-2020, with a sharper increase in Kuwaiti physicians and pharmacists. Importantly, the national–expatriate balance is cadre-specific rather than a uniform feature of the workforce. By 2022, Kuwaitis already formed the majority of dentists (approximately 75%) and administrators, and physicians were approaching parity (approximately 41% Kuwaiti), indicating a broadly balanced distribution in most cadres. The principal exception is nursing, where Kuwaitis constitute only approximately 5% of the workforce; this is the one cadre in which reliance on expatriate staff remains a genuine structural imbalance. Demographic challenges remain in the physician workforce, where non-Kuwaiti physicians still constitute the largest share, comprising a remarkable 59% of the total. Nevertheless, it is worth noting that the percentage of Kuwaitis remains higher than previous reports, at 37% in 2006 (52), reflecting ongoing efforts to nationalise the physicians’ workforce. Furthermore, physicians’ density increased to 2.6 per 1,000 in 2022 from 1.73 in 2006 (52), which is higher than other GCC countries, such as Bahrain (0.7) and Oman (1.9), but remains below Saudi Arabia (3.4), Qatar (3.0), and the UAE (2.9) (59). Furthermore, the composition of Kuwaitis in the dental workforce stands at 75%, indicating a sufficient supply and a balanced distribution, with a robust rate of 0.63 per 1,000, surpassing all GCC countries (60). In nursing, a different pattern emerged: Kuwaiti nurses’ recruitment declined pre-2020 but increased significantly post-2020, suggesting a national effort to nationalise the nursing workforce. However, the K nursing workforce still comprises only 5%, whereas the overall K share of nurses has decreased from 2011 (−37%); therefore, the reliance on expatriates remains high. Furthermore, nurses' density lags that of other GCC countries (61), such as Saudi Arabia, Qatar, and the UAE, highlighting a critical area for development.

### Services utilisation

The utilisation of healthcare services varied across Kuwaitis and non-Kuwaitis between 2011 and 2022. While the number of discharges for Kuwaiti patients increased in general hospitals (+9%) but fell in tertiary hospitals (−20%), discharges for non-Kuwaiti patients declined in both general (−8%) and tertiary hospitals (−36%). The non-Kuwaiti decline in general-hospital discharges is consistent with demographic shifts after 2020, driven by pandemic-induced regulations on expatriates and the outflow of the non-Kuwaiti workforce, with more than 100,000 expatriates reported to have left Kuwait in 2020 (62–65). The steeper tertiary-care declines in both populations, by contrast, reflect the curtailment of elective and non-urgent services at the 2020 pandemic onset, as capacity and staff were redirected toward the pandemic response, with discharge volumes not returning to pre-pandemic levels by 2022. GP visits dropped sharply at the 2020 pandemic onset for both Kuwaiti (*β*_2_ = −7.07 million; *p* < .001) and non-Kuwaiti patients (*β*_2_ = −2.32 million; *p* = .012), resembling a “systemic shock' due to the pandemic. However, Kuwaiti diabetes-care visits dropped significantly at the 2020 onset (*β*_2_ = −68,601; *p* = .006) but recovered significantly thereafter (*β*_3_ =  + 25,919/year; *p* = .012), and by the end of the period remained close to their pre-pandemic level, indicating a persistent burden of NCDs that resumed after the initial pandemic disruption (8). This pattern contrasts with international reports indicating significant declines in diabetes clinic visits and other outpatient services (62, 66). The latter explanation applies to the Kuwaiti context, in which many members of the dental workforce were reassigned to other services, such as COVID-19 testing centres or contact tracing teams (63).

Regarding vaccination uptake, mixed results make the overall trend very difficult to discern. For example, the influenza vaccine uptake among both Kuwaitis and non-Kuwaitis increased prior to 2020, highlighting the MOH's efforts to encourage seasonal vaccination. On the other hand, routine vaccinations, such as Hepatitis B and MMR, were either stable or declining pre-2020. Furthermore, most vaccinations have not shown a significant change in uptake post-2020, suggesting that the pandemic has disrupted vaccination delivery and delayed recovery in critical routine vaccinations. This is consistent with international observations of the pandemic's impact on vaccination rates and is also reflected in declines in children's vaccination rates (64). Nevertheless, Kuwait still shows high vaccination rates compared to international standards (65, 67, 68).

### Challenges and future directions

Despite significant achievements in Kuwait's health services and healthcare system from 2011 to 2022, challenges remain. Firstly, while the national–expatriate balance is reasonable across most cadres, the nursing workforce remains heavily dependent on expatriate staff (only approximately 5% Kuwaiti), and this specific imbalance needs to be addressed, a challenge shared across the GCC countries (22). Countries like Saudi Arabia have attempted to tackle this challenge by adopting nationalisation strategies (35), which set clear percentage targets for recruiting Saudi nationals across professions. Another challenge is the economic burden of NCDs on the healthcare system, as reflected in a 20% increase in diabetes-clinic visits among Kuwaitis and a 30% increase among non-Kuwaitis, suggesting the need for intervention through expanded preventive services. While it might not be a significant concern at present, the decline in vaccination uptake must be seen as a future challenge that can be addressed.

## Limitations

This study has several advantages; for instance, it relied on government-published data, thereby increasing the reliability and robustness of the results. Additionally, the study included indicators of the COVID-19 pandemic, demonstrating its effects on the healthcare system. However, several limitations are associated with the study. Firstly, concerning financial measures, it was difficult to discern information by age, income, or comorbidity profiles. Similarly, the economic data did not identify expenditure areas, leading to assumptions about high-cost areas for the MOH. Another limitation is that the post-2020 segment consists of 3 years only (2020–2022); this may explain the volatility in the utilisation results, since the last published statistic report by the MOH was for 2022. Nevertheless, aiming for a segmented regression analysis remains a robust method for detecting a structural break in this case (54). The dataset comprises *n* = 12 annual observations, with 9 pre-pandemic points (2011–2019) and only 3 post-pandemic points (2020–2022). This asymmetry inflates the confidence intervals and reduces statistical power for the post-pandemic slope-change parameter (*β*_3_). Accordingly, the immediate level shift at pandemic onset (*β*_2_), estimated from the full pre/post contrast, is the more robust inferential anchor, and the post-pandemic recovery slopes (*β*_3_) should be read as directional, lower-certainty estimates pending additional post-2022 data.

## Policy recommendations

Kuwait must adopt a targeted approach to recruiting Kuwaiti nurses to ensure workforce stability through the Kuwaitisation policy. The literature indicates that a coordinated, multi-dimensional strategy is necessary to enhance nurses' recruitment and retention, including improving working conditions (69, 70), expanding nursing school capacity (71, 72), and implementing workforce planning and evidence-based policy decisions (73, 74). Regarding vaccinations, several studies showed the effects of community education (75, 76), improved access and logistics, and reminders (77). However, combining multiple strategies has been shown to yield better results in increasing vaccination uptake (78, 79). In terms of NCD control policies, and as in other aspects, multi-sectoral policy approaches addressing key risk factors, including tobacco use, unhealthy diets, and physical inactivity (80–82). By focusing more on Kuwait studies, it was also shown that multifaceted policies can reduce NCDs and their burden in the community (9).

## Future studies

Future studies should explore in detail the effects of infrastructure expansion on the healthcare system in Kuwait, including utilisation and health indicators. Moreover, an in-depth analysis of the health workforce's status, demands, and production must be conducted to assist policymakers in making informed decisions about health workers. Additionally, a comprehensive health economic analysis of the K healthcare system is essential. Finally, the causes and consequences of the discrepancy in utilisation of the health services between Kuwaitis and non-Kuwaitis should be examined.

## Conclusion

This study demonstrated significant development in Kuwait's healthcare system between 2011 and 2022, particularly in infrastructure and expenditure. COVID-19 caused marked disruption to service utilisation, followed by substantial recovery across most services, consistent with system resilience. While workforce metrics are broadly comparable to those of regional systems, nursing remains heavily dependent on expatriate staff, and the rising burden of non-communicable diseases underscores the need for sustained investment in primary and preventive care. Targeted workforce and Kuwaitisation policies will be critical to long-term performance.

## Data Availability

Publicly available datasets were analyzed in this study. This data can be found here: data were aggregated from Kuwait's Ministry of Health annual reports.
