# Supplementary material for: Health system development and utilisation in Kuwait, 2011–2022: insights from national healthcare data
Source: Front Health Serv. 2026 Jul 17;6:1850596. doi: 10.3389/frhs.2026.1850596 (PMC13424385; doi:10.3389/frhs.2026.1850596)
Supplement: Supplementary file 1 [file Table1.docx]

*Table S1. Residual diagnostics and model-fit summary for the segmented OLS interrupted-time-series model, by indicator (n = 12 annual observations, 2011–2022).*

| **Indicator** | **Shapiro–Wilk W** | **Shapiro–Wilk p** | **Durbin–Watson** | **OLS AIC** | **OLS BIC** | **Max │stud. resid.│** | **Diagnostic flags** |
| --- | --- | --- | --- | --- | --- | --- | --- |
| ***Infrastructure*** | | | | | | | |
| Hospital beds | 0.889 | 0.110 | 2.10 | 115.6 | 117.5 | 2.31 | — |
| PHC centres | 0.952 | 0.660 | 1.53 | 17.9 | 19.8 | 2.30 | — |
| General & specialty hospitals | 0.832 | 0.022 | 1.50 | 10.8 | 12.7 | 2.05 | non-normal |
| Private hospitals | 0.876 | 0.077 | 1.50 | 6.0 | 7.9 | 2.81 | non-normal; AR(1) preferred |
| ***Expenditure*** | | | | | | | |
| Government health-sector salaries (M KWD) | 0.948 | 0.610 | 3.30 | 76.4 | 78.3 | 1.72 | AR(1) preferred |
| Drugs & equipment (M KWD) | 0.722 | 0.001 | 2.39 | 88.5 | 90.5 | 2.65 | non-normal |
| Budget executed (M KWD) | 0.977 | 0.970 | 2.31 | 132.8 | 134.7 | 2.02 | — |
| ***Hospital discharges*** | | | | | | | |
| General-hospital discharges, Kuwaiti | 0.844 | 0.031 | 1.90 | 190.9 | 192.8 | 1.76 | non-normal |
| General-hospital discharges, non-Kuwaiti | 0.926 | 0.340 | 2.03 | 185.9 | 187.9 | 1.63 | — |
| Tertiary discharges, Kuwaiti | 0.975 | 0.950 | 1.20 | 154.6 | 156.5 | 1.86 | AR(1) preferred |
| Tertiary discharges, non-Kuwaiti | 0.953 | 0.680 | 0.79 | 179.2 | 181.2 | 1.59 | AR(1) preferred |
| ***Primary-care visits*** | | | | | | | |
| GP visits, Kuwaiti | 0.949 | 0.630 | 3.19 | 303.2 | 305.1 | 1.49 | AR(1) preferred |
| GP visits, non-Kuwaiti | 0.956 | 0.730 | 0.83 | 339.5 | 341.5 | 1.34 | AR(1) preferred |
| Child-care visits, Kuwaiti | 0.968 | 0.890 | 2.84 | 282.7 | 284.7 | 1.92 | AR(1) preferred |
| Child-care visits, non-Kuwaiti | 0.942 | 0.530 | 1.59 | 286.9 | 288.8 | 2.20 | — |
| Dental visits, Kuwaiti | 0.912 | 0.230 | 2.53 | 244.0 | 245.9 | 2.16 | AR(1) preferred |
| Dental visits, non-Kuwaiti | 0.951 | 0.660 | 0.82 | 261.0 | 263.0 | 1.72 | AR(1) preferred |
| Diabetes-care visits, Kuwaiti | 0.979 | 0.980 | 1.20 | 226.9 | 228.9 | 2.16 | AR(1) preferred |
| Diabetes-care visits, non-Kuwaiti | 0.973 | 0.940 | 1.10 | 259.9 | 261.9 | 2.06 | AR(1) preferred |
| ***Vaccinations*** | | | | | | | |
| HepB vaccinations, Kuwaiti | 0.875 | 0.077 | 1.05 | 261.0 | 263.0 | 1.79 | borderline; AR(1) preferred |
| HepB vaccinations, non-Kuwaiti | 0.908 | 0.200 | 1.64 | 229.0 | 231.0 | 1.46 | — |
| Meningitis vaccinations, Kuwaiti | 0.910 | 0.214 | 2.22 | 212.4 | 214.3 | 2.08 | — |
| Meningitis vaccinations, non-Kuwaiti | 0.930 | 0.380 | 2.15 | 206.3 | 208.3 | 1.96 | — |
| Influenza vaccinations, Kuwaiti | 0.896 | 0.140 | 2.43 | 235.0 | 237.0 | 2.22 | — |
| Influenza vaccinations, non-Kuwaiti | 0.936 | 0.450 | 2.32 | 245.2 | 247.1 | 2.08 | — |
| MMR vaccinations, Kuwaiti | 0.803 | 0.010 | 2.73 | 188.0 | 189.9 | 2.62 | non-normal; AR(1) preferred |
| MMR vaccinations, non-Kuwaiti | 0.839 | 0.027 | 1.97 | 186.0 | 188.0 | 2.25 | non-normal |
| ***Workforce*** | | | | | | | |
| Physicians, Kuwaiti | 0.840 | 0.028 | 1.84 | 78.6 | 80.5 | 2.29 | non-normal |
| Physicians, non-Kuwaiti | 0.863 | 0.054 | 1.09 | 132.6 | 134.6 | 1.30 | non-normal; AR(1) preferred |
| Nurses, Kuwaiti | 0.910 | 0.210 | 2.19 | 73.2 | 75.2 | 2.15 | — |
| Nurses, non-Kuwaiti | 0.960 | 0.790 | 1.16 | 172.8 | 174.7 | 1.88 | AR(1) preferred |
| Pharmacists, Kuwaiti | 0.887 | 0.110 | 1.78 | 75.7 | 77.6 | 2.48 | — |
| Pharmacists, non-Kuwaiti | 0.917 | 0.260 | 1.74 | 102.5 | 104.4 | 1.80 | — |
| Dentists, Kuwaiti | 0.889 | 0.120 | 1.72 | 78.8 | 80.8 | 2.00 | — |
| Dentists, non-Kuwaiti | 0.820 | 0.016 | 1.73 | 73.1 | 75.1 | 1.57 | non-normal |
| Medical technicians, Kuwaiti | 0.950 | 0.640 | 2.69 | 102.1 | 104.0 | 2.10 | AR(1) preferred |
| Medical technicians, non-Kuwaiti | 0.945 | 0.570 | 1.91 | 132.1 | 134.0 | 2.00 | — |
| Administrators, Kuwaiti | 0.975 | 0.960 | 1.57 | 149.7 | 151.6 | 2.13 | — |
| Administrators, non-Kuwaiti | 0.906 | 0.190 | 2.33 | 109.0 | 111.0 | 2.40 | — |

*Table S2. Augmented Dickey–Fuller (ADF) stationarity tests for each indicator, in levels and first differences (n = 12 annual observations, 2011–2022).*

| **Indicator** | **ADF p (levels)** | **ADF p (Δ)** | **Classification (α = 0.05)** |
| --- | --- | --- | --- |
| ***Infrastructure*** | | | |
| Hospital beds | 0.998 | 0.062 | Non-stationary in levels and Δ |
| PHC centres | 0.999 | 0.015 | Stationary after first differencing (I(1)) |
| General & specialty hospitals | 0.817 | 0.202 | Non-stationary in levels and Δ |
| Private hospitals | <.001 | <.001 | Stationary in levels (I(0)) |
| ***Expenditure*** | | | |
| Government health-sector salaries (M KWD) | 0.083 | <.001 | Stationary after first differencing (I(1)) |
| Drugs & equipment (M KWD) | <.001 | <.001 | Stationary in levels (I(0)) |
| Budget executed (M KWD) | 1.000 | 0.988 | Non-stationary in levels and Δ |
| ***Hospital discharges*** | | | |
| General-hospital discharges, Kuwaiti | 0.947 | <.001 | Stationary after first differencing (I(1)) |
| General-hospital discharges, non-Kuwaiti | 1.000 | 0.987 | Non-stationary in levels and Δ |
| Tertiary discharges, Kuwaiti | <.001 | 0.005 | Stationary in levels (I(0)) |
| Tertiary discharges, non-Kuwaiti | 0.003 | 0.675 | Stationary in levels (I(0)) |
| ***Primary-care visits*** | | | |
| GP visits, Kuwaiti | 0.376 | 0.989 | Non-stationary in levels and Δ |
| GP visits, non-Kuwaiti | 0.000 | 0.020 | Stationary after first differencing (I(1)) |
| Child-care visits, Kuwaiti | 0.616 | 0.981 | Non-stationary in levels and Δ |
| Child-care visits, non-Kuwaiti | 1.000 | 0.007 | Stationary after first differencing (I(1)) |
| Dental visits, Kuwaiti | 0.247 | 0.034 | Stationary after first differencing (I(1)) |
| Dental visits, non-Kuwaiti | 0.164 | 0.008 | Stationary after first differencing (I(1)) |
| Diabetes-care visits, Kuwaiti | 0.984 | 0.004 | Stationary after first differencing (I(1)) |
| Diabetes-care visits, non-Kuwaiti | 0.168 | 0.054 | Non-stationary in levels and Δ |
| ***Vaccinations*** | | | |
| HepB vaccinations, Kuwaiti | 0.891 | <.001 | Stationary after first differencing (I(1)) |
| HepB vaccinations, non-Kuwaiti | 0.001 | 0.001 | Stationary in levels (I(0)) |
| Meningitis vaccinations, Kuwaiti | 0.065 | 0.628 | Non-stationary in levels and Δ |
| Meningitis vaccinations, non-Kuwaiti | 0.057 | 0.124 | Non-stationary in levels and Δ |
| Influenza vaccinations, Kuwaiti | 0.102 | <.001 | Stationary after first differencing (I(1)) |
| Influenza vaccinations, non-Kuwaiti | 0.845 | 0.062 | Non-stationary in levels and Δ |
| MMR vaccinations, Kuwaiti | 0.998 | <.001 | Stationary after first differencing (I(1)) |
| MMR vaccinations, non-Kuwaiti | 1.000 | 0.977 | Non-stationary in levels and Δ |
| ***Workforce*** | | | |
| Physicians, Kuwaiti | 1.000 | 0.994 | Non-stationary in levels and Δ |
| Physicians, non-Kuwaiti | 0.965 | 0.431 | Non-stationary in levels and Δ |
| Nurses, Kuwaiti | 0.496 | 0.070 | Non-stationary in levels and Δ |
| Nurses, non-Kuwaiti | 0.302 | 0.504 | Non-stationary in levels and Δ |
| Pharmacists, Kuwaiti | 0.999 | 0.997 | Non-stationary in levels and Δ |
| Pharmacists, non-Kuwaiti | 0.973 | 0.061 | Non-stationary in levels and Δ |
| Dentists, Kuwaiti | 1.000 | 0.998 | Non-stationary in levels and Δ |
| Dentists, non-Kuwaiti | 0.978 | 0.316 | Non-stationary in levels and Δ |
| Medical technicians, Kuwaiti | 1.000 | <.001 | Stationary after first differencing (I(1)) |
| Medical technicians, non-Kuwaiti | 0.918 | 0.045 | Stationary after first differencing (I(1)) |
| Administrators, Kuwaiti | 0.007 | 0.803 | Stationary in levels (I(0)) |
| Administrators, non-Kuwaiti | 0.103 | 0.500 | Non-stationary in levels and Δ |

*Table S3a. OLS segmented-regression coefficients for the interrupted-time-series model, all 39 indicators (n = 12 annual observations, 2011–2022).*

| **Variable** | **Parameter** | **B** | **SE** | **t** | **p** | **95% CI Lower** | **95% CI Upper** |
| --- | --- | --- | --- | --- | --- | --- | --- |
| Hospital beds | β₁ (pre-2020 trend) | 88.9 | 14.0 | 6.35 | <.001 | 56.6 | 121.1 |
|  | β₂ (level shift 2020) | 841.8 | 178.4 | 4.72 | 0.002 | 430.4 | 1,253 |
|  | β₃ (post-2020 slope Δ) | 90.1 | 77.9 | 1.16 | 0.280 | -89.4 | 269.7 |
| PHC centres | β₁ (pre-2020 trend) | 1.133 | 0.239 | 4.75 | 0.001 | 0.583 | 1.684 |
|  | β₂ (level shift 2020) | -9.644 | 3.043 | -3.17 | 0.013 | -16.7 | -2.628 |
|  | β₃ (post-2020 slope Δ) | 5.367 | 1.328 | 4.04 | 0.004 | 2.303 | 8.430 |
| General & specialty hospitals | β₁ (pre-2020 trend) | 0.750 | 0.178 | 4.22 | 0.003 | 0.340 | 1.160 |
|  | β₂ (level shift 2020) | 1.111 | 2.265 | 0.49 | 0.637 | -4.112 | 6.334 |
|  | β₃ (post-2020 slope Δ) | -0.750 | 0.989 | -0.76 | 0.470 | -3.030 | 1.530 |
| Private hospitals | β₁ (pre-2020 trend) | 0.267 | 0.145 | 1.84 | 0.104 | -0.068 | 0.602 |
|  | β₂ (level shift 2020) | -0.956 | 1.852 | -0.52 | 0.620 | -5.227 | 3.315 |
|  | β₃ (post-2020 slope Δ) | 0.233 | 0.809 | 0.29 | 0.780 | -1.631 | 2.098 |
| Govt health-sector salaries (M KWD) | β₁ (pre-2020 trend) | 61.2 | 2.734 | 22.38 | <.001 | 54.9 | 67.5 |
|  | β₂ (level shift 2020) | 95.8 | 34.9 | 2.75 | 0.025 | 15.4 | 176.2 |
|  | β₃ (post-2020 slope Δ) | -71.4 | 15.2 | -4.69 | 0.002 | -106.5 | -36.3 |
| Drugs & equipment (M KWD) | β₁ (pre-2020 trend) | 40.5 | 4.532 | 8.95 | <.001 | 30.1 | 51.0 |
|  | β₂ (level shift 2020) | -62.7 | 57.8 | -1.08 | 0.309 | -196.0 | 70.5 |
|  | β₃ (post-2020 slope Δ) | -18.6 | 25.2 | -0.74 | 0.483 | -76.8 | 39.6 |
| Budget executed (M KWD) | β₁ (pre-2020 trend) | 163.2 | 28.7 | 5.70 | <.001 | 97.2 | 229.3 |
|  | β₂ (level shift 2020) | -350.1 | 365.4 | -0.96 | 0.366 | -1,193 | 492.6 |
|  | β₃ (post-2020 slope Δ) | 234.9 | 159.5 | 1.47 | 0.179 | -133.0 | 602.7 |
| General-hospital discharges, Kuwaiti | β₁ (pre-2020 trend) | 684.6 | 322.0 | 2.13 | 0.066 | -58.1 | 1,427 |
|  | β₂ (level shift 2020) | -28,437 | 4,107 | -6.92 | <.001 | -37,908 | -18,965 |
|  | β₃ (post-2020 slope Δ) | 9,342 | 1,793 | 5.21 | <.001 | 5,208 | 13,477 |
| General-hospital discharges, non-Kuwaiti | β₁ (pre-2020 trend) | 254.5 | 262.1 | 0.97 | 0.360 | -349.9 | 859.0 |
|  | β₂ (level shift 2020) | -31,103 | 3,343 | -9.30 | <.001 | -38,813 | -23,394 |
|  | β₃ (post-2020 slope Δ) | 6,322 | 1,459 | 4.33 | 0.003 | 2,957 | 9,688 |
| Tertiary discharges, Kuwaiti | β₁ (pre-2020 trend) | 230.9 | 71.0 | 3.25 | 0.012 | 67.2 | 394.6 |
|  | β₂ (level shift 2020) | -9,766 | 905.3 | -10.79 | <.001 | -11,854 | -7,679 |
|  | β₃ (post-2020 slope Δ) | 1,123 | 395.2 | 2.84 | 0.022 | 211.2 | 2,034 |
| Tertiary discharges, non-Kuwaiti | β₁ (pre-2020 trend) | -441.9 | 198.5 | -2.23 | 0.057 | -899.7 | 15.8 |
|  | β₂ (level shift 2020) | -8,050 | 2,532 | -3.18 | 0.013 | -13,887 | -2,212 |
|  | β₃ (post-2020 slope Δ) | -136.1 | 1,105 | -0.12 | 0.905 | -2,685 | 2,412 |
| GP visits, Kuwaiti | β₁ (pre-2020 trend) | 355,406 | 34,717 | 10.24 | <.001 | 275,349 | 435,464 |
|  | β₂ (level shift 2020) | -7,071,803 | 442,785 | -15.97 | <.001 | -8,092,868 | -6,050,739 |
|  | β₃ (post-2020 slope Δ) | 1,397,378 | 193,297 | 7.23 | <.001 | 951,634 | 1,843,121 |
| GP visits, non-Kuwaiti | β₁ (pre-2020 trend) | 48,693 | 56,023 | 0.87 | 0.410 | -80,497 | 177,883 |
|  | β₂ (level shift 2020) | -2,319,953 | 714,525 | -3.25 | 0.012 | -3,967,651 | -672,255 |
|  | β₃ (post-2020 slope Δ) | 228,021 | 311,924 | 0.73 | 0.486 | -491,277 | 947,320 |
| Child-care visits, Kuwaiti | β₁ (pre-2020 trend) | 3,562 | 14,803 | 0.24 | 0.816 | -30,573 | 37,697 |
|  | β₂ (level shift 2020) | -2,574,075 | 188,796 | -13.63 | <.001 | -3,009,439 | -2,138,712 |
|  | β₃ (post-2020 slope Δ) | 541,468 | 82,418 | 6.57 | <.001 | 351,411 | 731,525 |
| Child-care visits, non-Kuwaiti | β₁ (pre-2020 trend) | -9,256 | 17,598 | -0.53 | 0.613 | -49,837 | 31,326 |
|  | β₂ (level shift 2020) | -773,221 | 224,448 | -3.44 | 0.009 | -1,290,799 | -255,643 |
|  | β₃ (post-2020 slope Δ) | 137,513 | 97,982 | 1.40 | 0.198 | -88,435 | 363,460 |
| Dental visits, Kuwaiti | β₁ (pre-2020 trend) | 7,956 | 2,949 | 2.70 | 0.027 | 1,155 | 14,756 |
|  | β₂ (level shift 2020) | -314,062 | 37,612 | -8.35 | <.001 | -400,796 | -227,329 |
|  | β₃ (post-2020 slope Δ) | 55,697 | 16,419 | 3.39 | 0.009 | 17,833 | 93,560 |
| Dental visits, non-Kuwaiti | β₁ (pre-2020 trend) | -1,897 | 5,994 | -0.32 | 0.760 | -15,720 | 11,926 |
|  | β₂ (level shift 2020) | -202,750 | 76,450 | -2.65 | 0.029 | -379,044 | -26,456 |
|  | β₃ (post-2020 slope Δ) | 53,101 | 33,374 | 1.59 | 0.150 | -23,859 | 130,062 |
| Diabetes visits, Kuwaiti | β₁ (pre-2020 trend) | 4,727 | 1,448 | 3.26 | 0.011 | 1,387 | 8,067 |
|  | β₂ (level shift 2020) | -68,601 | 18,472 | -3.71 | 0.006 | -111,198 | -26,005 |
|  | β₃ (post-2020 slope Δ) | 25,919 | 8,064 | 3.21 | 0.012 | 7,323 | 44,514 |
| Diabetes visits, non-Kuwaiti | β₁ (pre-2020 trend) | 20,773 | 5,723 | 3.63 | 0.007 | 7,575 | 33,972 |
|  | β₂ (level shift 2020) | -190,980 | 72,996 | -2.62 | 0.031 | -359,309 | -22,650 |
|  | β₃ (post-2020 slope Δ) | 9,496 | 31,866 | 0.30 | 0.773 | -63,988 | 82,980 |
| HepB vaccinations, Kuwaiti | β₁ (pre-2020 trend) | -5,246 | 1,446 | -3.63 | 0.007 | -8,580 | -1,912 |
|  | β₂ (level shift 2020) | 2,950 | 18,439 | 0.16 | 0.877 | -39,571 | 45,471 |
|  | β₃ (post-2020 slope Δ) | 6,461 | 8,050 | 0.80 | 0.445 | -12,102 | 25,023 |
| HepB vaccinations, non-Kuwaiti | β₁ (pre-2020 trend) | 397.8 | 381.1 | 1.04 | 0.327 | -481.0 | 1,277 |
|  | β₂ (level shift 2020) | -11,465 | 4,860 | -2.36 | 0.046 | -22,673 | -257.1 |
|  | β₃ (post-2020 slope Δ) | 2,328 | 2,122 | 1.10 | 0.304 | -2,565 | 7,221 |
| Meningitis vaccinations, Kuwaiti | β₁ (pre-2020 trend) | 1,109 | 789.6 | 1.40 | 0.198 | -711.9 | 2,930 |
|  | β₂ (level shift 2020) | 7,018 | 10,071 | 0.70 | 0.506 | -16,205 | 30,240 |
|  | β₃ (post-2020 slope Δ) | -6,866 | 4,396 | -1.56 | 0.157 | -17,004 | 3,272 |
| Meningitis vaccinations, non-Kuwaiti | β₁ (pre-2020 trend) | 1,008 | 613.9 | 1.64 | 0.139 | -407.4 | 2,424 |
|  | β₂ (level shift 2020) | 1,165 | 7,830 | 0.15 | 0.885 | -16,892 | 19,221 |
|  | β₃ (post-2020 slope Δ) | -5,706 | 3,418 | -1.67 | 0.134 | -13,589 | 2,177 |
| Influenza vaccinations, Kuwaiti | β₁ (pre-2020 trend) | 4,162 | 2,027 | 2.05 | 0.074 | -512.9 | 8,836 |
|  | β₂ (level shift 2020) | 22,890 | 25,854 | 0.89 | 0.402 | -36,730 | 82,511 |
|  | β₃ (post-2020 slope Δ) | -15,744 | 11,287 | -1.40 | 0.201 | -41,771 | 10,283 |
| Influenza vaccinations, non-Kuwaiti | β₁ (pre-2020 trend) | 12,237 | 3,094 | 3.96 | 0.004 | 5,103 | 19,371 |
|  | β₂ (level shift 2020) | -106,731 | 39,456 | -2.71 | 0.027 | -197,717 | -15,746 |
|  | β₃ (post-2020 slope Δ) | 41,184 | 17,224 | 2.39 | 0.044 | 1,465 | 80,904 |
| MMR vaccinations, Kuwaiti | β₁ (pre-2020 trend) | -572.1 | 285.6 | -2.00 | 0.080 | -1,231 | 86.6 |
|  | β₂ (level shift 2020) | -5,969 | 3,643 | -1.64 | 0.140 | -14,370 | 2,431 |
|  | β₃ (post-2020 slope Δ) | 892.6 | 1,590 | 0.56 | 0.590 | -2,775 | 4,560 |
| MMR vaccinations, non-Kuwaiti | β₁ (pre-2020 trend) | -24.5 | 263.3 | -0.09 | 0.928 | -631.6 | 582.7 |
|  | β₂ (level shift 2020) | -6,470 | 3,358 | -1.93 | 0.090 | -14,214 | 1,273 |
|  | β₃ (post-2020 slope Δ) | -3,226 | 1,466 | -2.20 | 0.059 | -6,606 | 154.9 |
| Physicians, Kuwaiti | β₁ (pre-2020 trend) | 168.5 | 2.991 | 56.34 | <.001 | 161.6 | 175.4 |
|  | β₂ (level shift 2020) | -20.8 | 38.2 | -0.55 | 0.600 | -108.8 | 67.2 |
|  | β₃ (post-2020 slope Δ) | 132.0 | 16.7 | 7.92 | <.001 | 93.6 | 170.4 |
| Physicians, non-Kuwaiti | β₁ (pre-2020 trend) | 225.8 | 28.4 | 7.94 | <.001 | 160.3 | 291.4 |
|  | β₂ (level shift 2020) | -562.4 | 362.8 | -1.55 | 0.160 | -1,399 | 274.2 |
|  | β₃ (post-2020 slope Δ) | 300.6 | 158.4 | 1.90 | 0.094 | -64.5 | 665.9 |
| Nurses, Kuwaiti | β₁ (pre-2020 trend) | -20.6 | 2.394 | -8.61 | <.001 | -26.1 | -15.1 |
|  | β₂ (level shift 2020) | -24.5 | 30.5 | -0.80 | 0.445 | -94.9 | 45.9 |
|  | β₃ (post-2020 slope Δ) | 42.6 | 13.3 | 3.20 | 0.013 | 11.9 | 73.3 |
| Nurses, non-Kuwaiti | β₁ (pre-2020 trend) | 1,004 | 151.7 | 6.62 | <.001 | 654.5 | 1,354 |
|  | β₂ (level shift 2020) | -2,820 | 1,935 | -1.46 | 0.183 | -7,282 | 1,642 |
|  | β₃ (post-2020 slope Δ) | -326.3 | 844.7 | -0.39 | 0.709 | -2,274 | 1,622 |
| Pharmacists, Kuwaiti | β₁ (pre-2020 trend) | 44.9 | 2.653 | 16.92 | <.001 | 38.8 | 51.0 |
|  | β₂ (level shift 2020) | -23.8 | 33.8 | -0.70 | 0.503 | -101.8 | 54.3 |
|  | β₃ (post-2020 slope Δ) | 63.6 | 14.8 | 4.31 | 0.003 | 29.6 | 97.7 |
| Pharmacists, non-Kuwaiti | β₁ (pre-2020 trend) | 39.9 | 8.104 | 4.93 | 0.001 | 21.2 | 58.6 |
|  | β₂ (level shift 2020) | -306.0 | 103.4 | -2.96 | 0.018 | -544.3 | -67.7 |
|  | β₃ (post-2020 slope Δ) | 208.6 | 45.1 | 4.62 | 0.002 | 104.5 | 312.6 |
| Dentists, Kuwaiti | β₁ (pre-2020 trend) | 90.2 | 3.022 | 29.83 | <.001 | 83.2 | 97.1 |
|  | β₂ (level shift 2020) | -75.6 | 38.5 | -1.96 | 0.086 | -164.4 | 13.3 |
|  | β₃ (post-2020 slope Δ) | 109.3 | 16.8 | 6.50 | <.001 | 70.5 | 148.1 |
| Dentists, non-Kuwaiti | β₁ (pre-2020 trend) | 16.8 | 2.385 | 7.04 | <.001 | 11.3 | 22.3 |
|  | β₂ (level shift 2020) | -56.8 | 30.4 | -1.87 | 0.099 | -126.9 | 13.4 |
|  | β₃ (post-2020 slope Δ) | 44.7 | 13.3 | 3.37 | 0.010 | 14.1 | 75.3 |
| Medical technicians, Kuwaiti | β₁ (pre-2020 trend) | 180.7 | 7.971 | 22.66 | <.001 | 162.3 | 199.0 |
|  | β₂ (level shift 2020) | 5.333 | 101.7 | 0.05 | 0.959 | -229.1 | 239.8 |
|  | β₃ (post-2020 slope Δ) | 81.8 | 44.4 | 1.84 | 0.102 | -20.5 | 184.2 |
| Medical technicians, non-Kuwaiti | β₁ (pre-2020 trend) | 115.7 | 27.8 | 4.16 | 0.003 | 51.5 | 179.8 |
|  | β₂ (level shift 2020) | -1,323 | 354.8 | -3.73 | 0.006 | -2,141 | -504.7 |
|  | β₃ (post-2020 slope Δ) | 1,029 | 154.9 | 6.64 | <.001 | 671.7 | 1,386 |
| Administrators, Kuwaiti | β₁ (pre-2020 trend) | 396.2 | 57.8 | 6.85 | <.001 | 262.9 | 529.6 |
|  | β₂ (level shift 2020) | -392.2 | 737.7 | -0.53 | 0.609 | -2,093 | 1,309 |
|  | β₃ (post-2020 slope Δ) | -838.8 | 322.0 | -2.60 | 0.031 | -1,581 | -96.1 |
| Administrators, non-Kuwaiti | β₁ (pre-2020 trend) | -25.2 | 10.6 | -2.37 | 0.045 | -49.8 | -0.712 |
|  | β₂ (level shift 2020) | -248.4 | 135.7 | -1.83 | 0.105 | -561.4 | 64.5 |
|  | β₃ (post-2020 slope Δ) | 274.2 | 59.2 | 4.63 | 0.002 | 137.6 | 410.9 |

*Table S3b. AR(1) GLS segmented-regression coefficients for the interrupted-time-series model, all 39 indicators (n = 12 annual observations, 2011–2022).*

| **Variable** | **Parameter** | **B** | **SE** | **t** | **p** | **95% CI Lower** | **95% CI Upper** |
| --- | --- | --- | --- | --- | --- | --- | --- |
| Hospital beds | β₁ (pre-2020 trend) | 89.1 | 14.2 | 6.29 | <.001 | 55.6 | 122.6 |
|  | β₂ (level shift 2020) | 845.6 | 189.7 | 4.46 | 0.003 | 397.0 | 1,294 |
|  | β₃ (post-2020 slope Δ) | 88.1 | 82.4 | 1.07 | 0.321 | -106.8 | 282.9 |
| PHC centres | β₁ (pre-2020 trend) | 1.224 | 0.314 | 3.89 | 0.006 | 0.481 | 1.967 |
|  | β₂ (level shift 2020) | -11.3 | 2.978 | -3.81 | 0.007 | -18.4 | -4.300 |
|  | β₃ (post-2020 slope Δ) | 5.685 | 1.399 | 4.06 | 0.005 | 2.376 | 8.994 |
| General & specialty hospitals | β₁ (pre-2020 trend) | 0.750 | 0.219 | 3.43 | 0.011 | 0.232 | 1.268 |
|  | β₂ (level shift 2020) | 0.699 | 2.311 | 0.30 | 0.771 | -4.767 | 6.165 |
|  | β₃ (post-2020 slope Δ) | -0.635 | 1.049 | -0.60 | 0.564 | -3.116 | 1.846 |
| Private hospitals | β₁ (pre-2020 trend) | 0.222 | 0.126 | 1.75 | 0.123 | -0.076 | 0.520 |
|  | β₂ (level shift 2020) | -1.037 | 1.895 | -0.55 | 0.601 | -5.519 | 3.445 |
|  | β₃ (post-2020 slope Δ) | 0.364 | 0.823 | 0.44 | 0.672 | -1.582 | 2.310 |
| Govt health-sector salaries (M KWD) | β₁ (pre-2020 trend) | 62.0 | 1.297 | 47.78 | <.001 | 58.9 | 65.1 |
|  | β₂ (level shift 2020) | 78.7 | 21.7 | 3.63 | 0.008 | 27.4 | 130.0 |
|  | β₃ (post-2020 slope Δ) | -68.6 | 9.654 | -7.10 | <.001 | -91.4 | -45.7 |
| Drugs & equipment (M KWD) | β₁ (pre-2020 trend) | 40.8 | 4.203 | 9.71 | <.001 | 30.9 | 50.7 |
|  | β₂ (level shift 2020) | -66.6 | 59.8 | -1.11 | 0.302 | -208.1 | 74.8 |
|  | β₃ (post-2020 slope Δ) | -17.4 | 25.9 | -0.67 | 0.522 | -78.7 | 43.8 |
| Budget executed (M KWD) | β₁ (pre-2020 trend) | 162.8 | 25.2 | 6.45 | <.001 | 103.1 | 222.4 |
|  | β₂ (level shift 2020) | -260.0 | 369.9 | -0.70 | 0.505 | -1,135 | 614.7 |
|  | β₃ (post-2020 slope Δ) | 190.7 | 160.3 | 1.19 | 0.273 | -188.4 | 569.9 |
| General-hospital discharges, Kuwaiti | β₁ (pre-2020 trend) | 686.0 | 340.8 | 2.01 | 0.084 | -119.9 | 1,492 |
|  | β₂ (level shift 2020) | -28,415 | 4,390 | -6.47 | <.001 | -38,797 | -18,032 |
|  | β₃ (post-2020 slope Δ) | 9,325 | 1,914 | 4.87 | 0.002 | 4,798 | 13,852 |
| General-hospital discharges, non-Kuwaiti | β₁ (pre-2020 trend) | 269.7 | 254.0 | 1.06 | 0.324 | -331.0 | 870.4 |
|  | β₂ (level shift 2020) | -31,791 | 3,526 | -9.02 | <.001 | -40,130 | -23,451 |
|  | β₃ (post-2020 slope Δ) | 6,489 | 1,528 | 4.25 | 0.004 | 2,875 | 10,103 |
| Tertiary discharges, Kuwaiti | β₁ (pre-2020 trend) | 244.5 | 110.9 | 2.21 | 0.063 | -17.7 | 506.8 |
|  | β₂ (level shift 2020) | -10,545 | 745.9 | -14.14 | <.001 | -12,309 | -8,781 |
|  | β₃ (post-2020 slope Δ) | 1,220 | 400.1 | 3.05 | 0.019 | 273.8 | 2,166 |
| Tertiary discharges, non-Kuwaiti | β₁ (pre-2020 trend) | -405.5 | 295.0 | -1.38 | 0.212 | -1,103 | 292.2 |
|  | β₂ (level shift 2020) | -6,712 | 1,810 | -3.71 | 0.008 | -10,993 | -2,431 |
|  | β₃ (post-2020 slope Δ) | -372.5 | 1,008 | -0.37 | 0.723 | -2,756 | 2,011 |
| GP visits, Kuwaiti | β₁ (pre-2020 trend) | 356,903 | 19,603 | 18.21 | <.001 | 310,542 | 403,265 |
|  | β₂ (level shift 2020) | -7,041,571 | 322,441 | -21.84 | <.001 | -7,804,144 | -6,278,999 |
|  | β₃ (post-2020 slope Δ) | 1,355,458 | 142,632 | 9.50 | <.001 | 1,018,134 | 1,692,782 |
| GP visits, non-Kuwaiti | β₁ (pre-2020 trend) | 58,790 | 84,788 | 0.69 | 0.510 | -141,734 | 259,315 |
|  | β₂ (level shift 2020) | -1,883,514 | 512,508 | -3.67 | 0.008 | -3,095,595 | -671,433 |
|  | β₃ (post-2020 slope Δ) | 163,053 | 287,044 | 0.57 | 0.588 | -515,806 | 841,911 |
| Child-care visits, Kuwaiti | β₁ (pre-2020 trend) | 5,386 | 10,829 | 0.50 | 0.634 | -20,224 | 30,996 |
|  | β₂ (level shift 2020) | -2,620,051 | 169,587 | -15.45 | <.001 | -3,021,125 | -2,218,977 |
|  | β₃ (post-2020 slope Δ) | 549,475 | 74,031 | 7.42 | <.001 | 374,391 | 724,559 |
| Child-care visits, non-Kuwaiti | β₁ (pre-2020 trend) | -8,299 | 20,502 | -0.41 | 0.698 | -56,787 | 40,189 |
|  | β₂ (level shift 2020) | -755,251 | 236,621 | -3.19 | 0.015 | -1,314,859 | -195,643 |
|  | β₃ (post-2020 slope Δ) | 131,256 | 105,134 | 1.25 | 0.252 | -117,385 | 379,896 |
| Dental visits, Kuwaiti | β₁ (pre-2020 trend) | 7,292 | 2,472 | 2.95 | 0.021 | 1,447 | 13,137 |
|  | β₂ (level shift 2020) | -303,087 | 37,073 | -8.18 | <.001 | -390,765 | -215,409 |
|  | β₃ (post-2020 slope Δ) | 53,644 | 16,092 | 3.33 | 0.013 | 15,586 | 91,701 |
| Dental visits, non-Kuwaiti | β₁ (pre-2020 trend) | 862.1 | 8,636 | 0.10 | 0.923 | -19,563 | 21,287 |
|  | β₂ (level shift 2020) | -189,579 | 60,055 | -3.16 | 0.016 | -331,609 | -47,550 |
|  | β₃ (post-2020 slope Δ) | 45,178 | 31,779 | 1.42 | 0.198 | -29,980 | 120,335 |
| Diabetes visits, Kuwaiti | β₁ (pre-2020 trend) | 3,830 | 2,240 | 1.71 | 0.131 | -1,468 | 9,128 |
|  | β₂ (level shift 2020) | -50,848 | 14,484 | -3.51 | 0.010 | -85,101 | -16,594 |
|  | β₃ (post-2020 slope Δ) | 24,575 | 7,896 | 3.11 | 0.017 | 5,902 | 43,248 |
| Diabetes visits, non-Kuwaiti | β₁ (pre-2020 trend) | 17,462 | 9,121 | 1.91 | 0.097 | -4,111 | 39,034 |
|  | β₂ (level shift 2020) | -113,651 | 51,492 | -2.21 | 0.063 | -235,428 | 8,127 |
|  | β₃ (post-2020 slope Δ) | 6,060 | 29,615 | 0.20 | 0.844 | -63,980 | 76,100 |
| HepB vaccinations, Kuwaiti | β₁ (pre-2020 trend) | -6,077 | 2,297 | -2.65 | 0.033 | -11,509 | -645.3 |
|  | β₂ (level shift 2020) | -5,971 | 15,863 | -0.38 | 0.718 | -43,486 | 31,544 |
|  | β₃ (post-2020 slope Δ) | 9,278 | 8,417 | 1.10 | 0.307 | -10,629 | 29,185 |
| HepB vaccinations, non-Kuwaiti | β₁ (pre-2020 trend) | 357.9 | 452.7 | 0.79 | 0.455 | -712.7 | 1,428 |
|  | β₂ (level shift 2020) | -10,957 | 5,061 | -2.17 | 0.067 | -22,926 | 1,012 |
|  | β₃ (post-2020 slope Δ) | 2,299 | 2,265 | 1.01 | 0.344 | -3,058 | 7,656 |
| Meningitis vaccinations, Kuwaiti | β₁ (pre-2020 trend) | 976.1 | 718.2 | 1.36 | 0.216 | -722.4 | 2,675 |
|  | β₂ (level shift 2020) | 11,391 | 10,403 | 1.10 | 0.310 | -13,212 | 35,994 |
|  | β₃ (post-2020 slope Δ) | -8,288 | 4,507 | -1.84 | 0.109 | -18,947 | 2,371 |
| Meningitis vaccinations, non-Kuwaiti | β₁ (pre-2020 trend) | 925.0 | 594.7 | 1.56 | 0.164 | -481.5 | 2,331 |
|  | β₂ (level shift 2020) | 3,234 | 8,260 | 0.39 | 0.707 | -16,301 | 22,769 |
|  | β₃ (post-2020 slope Δ) | -6,332 | 3,580 | -1.77 | 0.120 | -14,798 | 2,134 |
| Influenza vaccinations, Kuwaiti | β₁ (pre-2020 trend) | 4,578 | 1,607 | 2.85 | 0.025 | 777.9 | 8,378 |
|  | β₂ (level shift 2020) | 21,689 | 24,739 | 0.88 | 0.410 | -36,818 | 80,196 |
|  | β₃ (post-2020 slope Δ) | -17,665 | 10,770 | -1.64 | 0.145 | -43,135 | 7,805 |
| Influenza vaccinations, non-Kuwaiti | β₁ (pre-2020 trend) | 12,779 | 2,782 | 4.59 | 0.003 | 6,201 | 19,358 |
|  | β₂ (level shift 2020) | -112,816 | 40,376 | -2.79 | 0.027 | -208,305 | -17,327 |
|  | β₃ (post-2020 slope Δ) | 41,479 | 17,494 | 2.37 | 0.050 | 107.2 | 82,852 |
| MMR vaccinations, Kuwaiti | β₁ (pre-2020 trend) | -669.7 | 202.2 | -3.31 | 0.013 | -1,148 | -191.5 |
|  | β₂ (level shift 2020) | -4,342 | 3,212 | -1.35 | 0.219 | -11,940 | 3,255 |
|  | β₃ (post-2020 slope Δ) | 387.3 | 1,406 | 0.28 | 0.791 | -2,939 | 3,714 |
| MMR vaccinations, non-Kuwaiti | β₁ (pre-2020 trend) | -62.8 | 254.2 | -0.25 | 0.812 | -663.9 | 538.3 |
|  | β₂ (level shift 2020) | -6,146 | 3,536 | -1.74 | 0.126 | -14,510 | 2,217 |
|  | β₃ (post-2020 slope Δ) | -3,286 | 1,532 | -2.14 | 0.069 | -6,910 | 338.3 |
| Physicians, Kuwaiti | β₁ (pre-2020 trend) | 168.5 | 2.972 | 56.71 | <.001 | 161.5 | 175.6 |
|  | β₂ (level shift 2020) | -14.7 | 40.5 | -0.36 | 0.727 | -110.5 | 81.1 |
|  | β₃ (post-2020 slope Δ) | 129.6 | 17.6 | 7.38 | <.001 | 88.1 | 171.2 |
| Physicians, non-Kuwaiti | β₁ (pre-2020 trend) | 224.7 | 41.6 | 5.40 | 0.001 | 126.2 | 323.1 |
|  | β₂ (level shift 2020) | -345.5 | 304.5 | -1.13 | 0.294 | -1,066 | 374.8 |
|  | β₃ (post-2020 slope Δ) | 269.8 | 157.8 | 1.71 | 0.131 | -103.5 | 643.0 |
| Nurses, Kuwaiti | β₁ (pre-2020 trend) | -20.6 | 2.366 | -8.71 | <.001 | -26.2 | -15.0 |
|  | β₂ (level shift 2020) | -26.3 | 32.3 | -0.81 | 0.443 | -102.6 | 50.1 |
|  | β₃ (post-2020 slope Δ) | 43.2 | 14.0 | 3.08 | 0.018 | 10.0 | 76.3 |
| Nurses, non-Kuwaiti | β₁ (pre-2020 trend) | 884.9 | 218.0 | 4.06 | 0.005 | 369.3 | 1,400 |
|  | β₂ (level shift 2020) | -1,461 | 1,603 | -0.91 | 0.393 | -5,252 | 2,331 |
|  | β₃ (post-2020 slope Δ) | -413.3 | 829.2 | -0.50 | 0.633 | -2,374 | 1,548 |
| Pharmacists, Kuwaiti | β₁ (pre-2020 trend) | 45.1 | 3.056 | 14.75 | <.001 | 37.8 | 52.3 |
|  | β₂ (level shift 2020) | -31.3 | 35.9 | -0.87 | 0.413 | -116.2 | 53.7 |
|  | β₃ (post-2020 slope Δ) | 65.6 | 15.9 | 4.12 | 0.004 | 28.0 | 103.2 |
| Pharmacists, non-Kuwaiti | β₁ (pre-2020 trend) | 39.6 | 9.255 | 4.28 | 0.004 | 17.8 | 61.5 |
|  | β₂ (level shift 2020) | -298.9 | 109.5 | -2.73 | 0.029 | -557.8 | -40.1 |
|  | β₃ (post-2020 slope Δ) | 205.6 | 48.4 | 4.25 | 0.004 | 91.2 | 320.1 |
| Dentists, Kuwaiti | β₁ (pre-2020 trend) | 90.0 | 3.081 | 29.21 | <.001 | 82.7 | 97.3 |
|  | β₂ (level shift 2020) | -75.3 | 41.1 | -1.83 | 0.109 | -172.5 | 21.8 |
|  | β₃ (post-2020 slope Δ) | 109.3 | 17.8 | 6.12 | <.001 | 67.1 | 151.5 |
| Dentists, non-Kuwaiti | β₁ (pre-2020 trend) | 16.8 | 2.729 | 6.15 | <.001 | 10.3 | 23.2 |
|  | β₂ (level shift 2020) | -55.1 | 32.2 | -1.71 | 0.131 | -131.2 | 21.0 |
|  | β₃ (post-2020 slope Δ) | 43.9 | 14.2 | 3.08 | 0.018 | 10.2 | 77.5 |
| Medical technicians, Kuwaiti | β₁ (pre-2020 trend) | 178.4 | 5.190 | 34.37 | <.001 | 166.1 | 190.6 |
|  | β₂ (level shift 2020) | 59.1 | 84.1 | 0.70 | 0.505 | -139.7 | 257.9 |
|  | β₃ (post-2020 slope Δ) | 74.4 | 37.0 | 2.01 | 0.084 | -13.1 | 161.9 |
| Medical technicians, non-Kuwaiti | β₁ (pre-2020 trend) | 115.7 | 29.7 | 3.90 | 0.006 | 45.5 | 185.8 |
|  | β₂ (level shift 2020) | -1,324 | 379.3 | -3.49 | 0.010 | -2,221 | -426.7 |
|  | β₃ (post-2020 slope Δ) | 1,029 | 165.5 | 6.22 | <.001 | 637.8 | 1,421 |
| Administrators, Kuwaiti | β₁ (pre-2020 trend) | 374.0 | 74.3 | 5.03 | 0.002 | 198.2 | 549.8 |
|  | β₂ (level shift 2020) | -52.2 | 736.6 | -0.07 | 0.946 | -1,794 | 1,690 |
|  | β₃ (post-2020 slope Δ) | -900.1 | 341.0 | -2.64 | 0.033 | -1,707 | -93.6 |
| Administrators, non-Kuwaiti | β₁ (pre-2020 trend) | -27.3 | 8.268 | -3.30 | 0.013 | -46.8 | -7.724 |
|  | β₂ (level shift 2020) | -265.0 | 128.7 | -2.06 | 0.079 | -569.5 | 39.5 |
|  | β₃ (post-2020 slope Δ) | 293.8 | 56.1 | 5.23 | 0.001 | 161.0 | 426.6 |

*Table S4. Bootstrap BCa 95 % confidence intervals (1,000 replicates) for indicators with Shapiro–Wilk p < .10.*

| **Indicator** | **Parameter** | **B** | **BCa 95% lower** | **BCa 95% upper** | **Notes** |
| --- | --- | --- | --- | --- | --- |
| ***Infrastructure*** | | | | | |
| General & specialty hospitals | β₁ Time | 0.750 | 0.269 | 1.059 |  |
|  | β₂ Intervention | 1.111 | 0.045 | 5.370 |  |
|  | β₃ Time_after | -0.750 | -1.236 | 0.000 |  |
| Private hospitals | β₁ Time | 0.267 | 0.000 | 0.768 |  |
|  | β₂ Intervention | -0.956 | -2.889 | 1.000 |  |
|  | β₃ Time_after | 0.233 | -0.469 | 1.000 |  |
| ***Expenditure*** | | | | | |
| Drugs & equipment (M KWD) | β₁ Time | 40.5 | 35.6 | 52.1 |  |
|  | β₂ Intervention | -62.7 | -144.2 | -3.469 |  |
|  | β₃ Time_after | -18.6 | -36.5 | -2.079 |  |
| ***Hospital discharges*** | | | | | |
| General-hospital discharges, Kuwaiti | β₁ Time | 684.6 | -438.0 | 2,066.3 |  |
|  | β₂ Intervention | -28,437 | -43,987 | -18,175 |  |
|  | β₃ Time_after | 9,342.4 | 3,654.6 | 14,944 |  |
| ***Vaccinations*** | | | | | |
| HepB vaccinations, Kuwaiti | β₁ Time | -5,246 | -8,868 | -1,448 |  |
|  | β₂ Intervention | 2,950 | -7,092 | 26,899 |  |
|  | β₃ Time_after | 6,461 | 2,398 | 10,961 |  |
| MMR vaccinations, Kuwaiti | β₁ Time | -572.1 | -1,439.2 | -98.2 |  |
|  | β₂ Intervention | -5,969.3 | -13,529 | -648.0 |  |
|  | β₃ Time_after | 892.6 | -1,502.8 | 3,384.1 |  |
| MMR vaccinations, non-Kuwaiti | β₁ Time | -24.5 | -854.7 | 618.4 |  |
|  | β₂ Intervention | -6,470.1 | -12,783 | -1,892.1 |  |
|  | β₃ Time_after | -3,225.5 | -5,333.6 | -1,040.7 |  |
| ***Workforce*** | | | | | |
| Physicians, Kuwaiti | β₁ Time | 168.5 | 162.2 | 175.2 |  |
|  | β₂ Intervention | -20.8 | -123.2 | 47.7 |  |
|  | β₃ Time_after | 132.0 | 96.4 | 167.9 |  |
| Physicians, non-Kuwaiti | β₁ Time | 225.8 | 135.4 | 330.2 |  |
|  | β₂ Intervention | -562.4 | -1,514.6 | -102.5 |  |
|  | β₃ Time_after | 300.6 | 19.4 | 556.1 |  |
| Dentists, non-Kuwaiti | β₁ Time | 16.8 | 12.8 | 22.5 |  |
|  | β₂ Intervention | -56.8 | -128.0 | 48.6 |  |
|  | β₃ Time_after | 44.7 | 7.842 | 81.4 |  |

*Bootstrap bias-corrected and accelerated (BCa) 95% confidence intervals are based on 1,000 replicates, computed for indicators with non-normal or borderline OLS residuals (Shapiro–Wilk p < .10). For some indicators, fewer valid replicates were available where resampled predictors became constant in small-sample draws; and the percentile method was used instead. Bounds reported as 0.000 correspond to values indistinguishable from zero.*
